# Runx3 Induces a Cell Shape Change and Suppresses Migration and Metastasis of Melanoma Cells by Altering a Transcriptional Profile

**DOI:** 10.3390/ijms22042219

**Published:** 2021-02-23

**Authors:** Ning Wang, Haiying Zhang, Xiulin Cui, Chao Ma, Linghui Wang, Wenguang Liu

**Affiliations:** 1 Institute of Genetics and Cell Biology, School of Life Sciences, Northeast Normal University, No. 5268, Renmin St., Changchun 130024, China; wangn657@nenu.edu.cn (N.W.); cuixl895@nenu.edu.cn (X.C.); mac420@nenu.edu.cn (C.M.); lwgqj@hotmail.com (L.W.); 2 Key Laboratory of Pathobiology of Ministry of Education, Norman Bethune College of Medicine, Jilin University, No. 126, Xinmin St., Changchun 130021, China; zhanghaiy@jlu.edu.cn

**Keywords:** Runx3, melanoma, metastasis, extracellular matrix, adhesion, actin cytoskeleton, transcriptional profile, *Mal*

## Abstract

Runt-related transcription factor-3 (Runx3) is a tumor suppressor, and its contribution to melanoma progression remains unclear. We previously demonstrated that Runx3 re-expression in B16-F10 melanoma cells changed their shape and attenuated their migration. In this study, we found that Runx3 re-expression in B16-F10 cells also suppressed their pulmonary metastasis. We performed microarray analysis and uncovered an altered transcriptional profile underlying the cell shape change and the suppression of migration and metastasis. This altered transcriptional profile was rich in Gene Ontology/Kyoto Encyclopedia of Genes and Genomes (GO/KEGG) annotations relevant to adhesion and the actin cytoskeleton and included differentially expressed genes for some major extracellular matrix (ECM) proteins as well as genes that were inversely associated with the increase in the metastatic potential of B16-F10 cells compared to B16-F0 melanoma cells. Further, we found that this altered transcriptional profile could have prognostic value, as evidenced by myelin and lymphocyte protein (*MAL*) and vilin-like (*VILL*). Finally, *Mal* gene expression was correlated with metastatic potential among the cells and was targeted by histone deacetylase (HDAC) inhibitors in B16-F10 cells, and the knockdown of *Mal* gene expression in B16-F0 cells changed their shape and enhanced the migratory and invasive traits of their metastasis. Our study suggests that self-entrapping of metastatic Runx3-negative melanoma cells via adhesion and the actin cytoskeleton could be a powerful therapeutic strategy.

## 1. Introduction

Cancer results in more than 8 million deaths every year worldwide, and cancer metastasis is the most frequent cause of these deaths, accounting for over 90% of mortalities [1,2]. Metastasis is hard to cure through current therapeutic regimens due to its systemic and complex nature and drug resistance [1,3,4,5,6]. As the molecular and cellular mechanisms involved in metastasis are still poorly understood, the discovery and development of effective treatments of cancer have been obstructed [3,5,6,7,8].

Cancer metastasis is a multistep cellular process; it involves cancer cell invasion, intravasation, survival in the circulation, arrest within the capillary bed, extravasation, and colonization at a distant site [1,3,7,8]. Along with this process, the local and foreign microenvironment, angiogenesis, platelet interaction, immunosurveillance, etc., together with many intrinsic characteristics of cancer cells, such as the epithelial–mesenchymal transition (EMT), energy metabolism, protease secretion, migratory capacity, exosome production, stemness, dormancy, and epi-/genetic drivers, affect successful metastasis [1,5,7,8]. Therefore, this process could provide unlimited candidates for various therapeutic targets [1,7,8,9].

Metastatic cancer cells can adapt their shape to be suitable for migration and invasion according to both environmental and genetic cues [5,10,11,12,13,14,15]. This cell shape change is a reflection of the dynamics in adhesion and the cytoskeleton, as well as other molecular and cellular processes [16]. Both of these, as well as the interplay between them, determine cancer cells’ migratory capacity [1,17,18,19], so these two evolutionarily conserved cellular processes could be therapeutically targeted for the treatment of metastasis [7,20]. The specific mechanisms that determine favorable cell shape changes for cancer cell migration and invasion during metastasis are not completely understood. Thus, the identification and characterization of key factors that can regulate cancer cells’ morphology would provide new insights into the pathology of metastasis [13].

The extracellular matrix (ECM) is a complex architecture that is capable of remodeling, and it supports cancer cell proliferation, survival, adhesion, migration, and invasion [21,22]. Many early and recent studies have thoroughly elucidated the cancer biology of individual ECM proteins or their genes, and a number of candidates for metastasis markers and therapeutic targets have been identified [23,24,25,26,27,28,29,30,31,32,33]. Although ECM proteins are very important in cancer metastasis, they remain underexplored [29,34].

The transcription factor runt-related transcription factor-3 (Runx3) plays a pivotal role in neurogenesis and thymopoiesis [35]. The depletion of Runx3 is a cause of gastric cancer development, and evidence that inactivation of Runx3 expression or function is a key event in gastric cancer and many other types of cancer has accumulated [36,37,38,39]. Loss of Runx3 expression has also frequently been found in melanoma cells as compared to melanocytes, and there is a good negative correlation between Runx3 expression and melanoma malignancy [40]. In addition, many functional studies have demonstrated that Runx3 is a tumor suppressor in many types of cancers but is a tumor promoter in other types [37,38,39,41,42,43,44].

The mode of action of Runx3 in the suppression of tumor progression is complex, but evidence shows that Runx3 governs the EMT during tumor progression [45,46,47,48]. The morphological changes in breast cancer cells are reminiscent of back–epithelial transitions and are closely accompanied by predominantly decreased Matrigel invasion after an increase in exogenous Runx3 expression [36]. In metastatic melanoma cells, a number of genes, including the tumor suppressor *PTEN*, were recently identified with a screen for morphological complexity as functionally conserved cell shape regulators that are relevant to disease progression [13]. Like PTEN, Runx3 may also function as a cell shape regulator when it suppresses melanoma cell migration and invasion in vitro [49]. Recently, additional roles of Runx3 in metastasis have been implicated in processes such as regulation of the cancer microenvironment, angiogenesis, stemness, immunosurveillance, and inflammation [42,48,50,51,52].

Myelin and lymphocyte protein (Mal) is a four-time transmembrane protein, and it plays a role in adhesion and the actin cytoskeleton [53,54]. Mal is also a tumor suppressor [53,55], and it may be involved in suppressing metastasis [56]. As a transcription factor, Runx3 has to fulfill its anti-metastasis function via its downstream genes. Whether Mal can be a therapeutic target for the treatment of metastasis is potentially interesting.

In this study, we used a metastatic cell model of mouse melanoma to further investigate the role of Runx3 in metastasis and to uncover the underlying transcriptional profile. We hope that this study can help in the discovery of therapeutic targets for the treatment of metastasis.

## 2. Materials and Methods

### 2.1. Generation of Stable Cell Lines by Lentiviral Infection

For the mock control cell line, transfection was performed with the empty lentiviral construct PLKO.1 and the packaging constructs psPAX2 and pMD.2G using the Lipofectamine 2000 transfection reagent (11668019, Invitrogen, Carlsbad, CA, USA). The viral supernatant was harvested at 48 h and filtered through a 0.45 µm filter. For the stable knockdown cell line for *Mal*, lentiviral particles were purchased from Santa Cruz (sc-44786-v, Mal shRNA (m)). B16-F0 mouse melanoma cells were infected with the lentiviral supernatants with the aid of 5 µg/mL of polybrene. All stable colonies were selected through treatment with 5 µg/mL of puromycin. The success of the knockdown of *Mal* gene expression in puromycin-resistant colonies was confirmed by RT-qPCR and Western blotting.

### 2.2. Cell Culture and Treatment, Cell Proliferation Assay, and Cell Cycle Assay

The generation of the Runx3-re-expressing B16-F10 melanoma cell line and the cultures of all the different melanoma cell lines in this study (B16-F10, mock control B16-F10, B16-F0, mock control B16-F0, B16-F10/Runx3, and B16-F0/shMal) have been previously described [49]. B16-F10 has high pulmonary metastatic ability, whereas B16-F0 is the parent cell line of B16-F10 and its pulmonary metastatic ability is low [57,58]. Histone deacetylase (HDAC) inhibitors (S1095, S2132, S2818, S2244, and S8464) were purchased from Selleck, and treatment of cells with an individual inhibitor was maintained for 18 h at 10 μM. The cell proliferation assay and the cell cycle assay were performed, as previously described, using the MTT method and flow cytometry for PI (Propidium Iodide) (Cat. No. BD5012, Bioworld)-stained cells, respectively [49,59].

### 2.3. Wound-Healing Assay and Transwell Migration/Invasion Assay

To address their migratory potential, cells were grown to 100% confluence in a culture medium containing a normal concentration of serum (10%). At hour 0, a 0.5-mm-wide wound was made with a rubber scrapper, and the detached cells were washed off with phosphate-buffered saline (PBS) buffer. The remaining cells were maintained in a medium supplemented with 1% serum for 48 h. During this period, cells were allowed to migrate into the wounded area. Transwell migration and invasion assays were performed, as previously described [49], using Costar Transwell chambers (Cat. No. 3422, Corning) with or without a Matrigel (Cat. No. 356234, Corning) coating. In brief, 10e5 cells were seeded on the upper part of the chamber with a medium containing 1% FBS, and the lower part of the chamber was incubated with a medium containing 10% FBS. Migrated or invaded cells on the membrane were stained with crystal violet.

### 2.4. Subcutaneous Tumor Formation and Pulmonary Metastasis Assay

C57BL/6J mice were bought from the Animal Experimental Center of Jilin University (Changchun, China). All mice were housed in a pathogen-free and air-conditioned environment with light cycles of 12 h on and 12 h off and free access to tap water and food. The starting age of experimental mice was 6–10 weeks old. Where applicable, mice were anesthetized. When assessing the tumor volume, a diameter of less than or around 1.0 cm per tumor was used. Tumor formation was generated by subcutaneous injection of 10e6 cells per mouse, and the tumors were allowed to grow for 3 weeks. The mice were sacrificed, and the tumors were carefully harvested. To detect pulmonary metastasis, 10e5 cells were intravenously injected per mouse, and the mice were sacrificed to record the metastasis foci in the lungs at 3 or 4 weeks after injection.

### 2.5. Microarray

SmartArray microarray chips (CapitalBio, Beijing, China) were prepared from 32K Mouse Genome Array version 4.0 (http://www.Operon.com, accessed on 25 April 2011). Total RNA was prepared using Trizol reagent (15596026, Invitrogen, ThermoFisher, Waltham, MA, USA) and was purified using the NucleoSpin RNA Clean-up kit (Macherey-Nagel, Düren, Germany). cRNA was amplified and labeled with Cy5- or Cy3-dCTP using the Jingxin cRNA amplification and labeling kit (CapitalBio, Beijing, China) after the synthesis of double-stranded cDNA. Hybridization on chips was performed in a hybridization buffer (3× SSC, 0.2% SDS, 5× Denhart’s, 25% formamide) at 42 °C overnight. After washing, the chips were scanned using a LuxScan 10 KA scanner, and the signals were analyzed using LuxScan 3.0 software (CapitalBio, Beijing, China). Differentially expressed genes from the comparative analysis were picked with a 0.5- or 2-fold threshold. Pathway and Gene Ontology (GO) annotations were performed using Molecule Annotation System version 3.0 (CapitalBio, Beijing, China).

### 2.6. RNA Preparation and RT-qPCR

Total RNA was extracted using Trizol reagent. Reverse transcription from the RNA was performed using the Takara PrimeScript RT reagent kit with gDNA Eraser (RR047A, Takara, Dalian, China). qPCR was performed using the 2× SYBR Green qPCR Master Mix (B21202, Bimake, Shanghai, China), and the PCR protocol was 95 °C for 15 s and 60 °C for 1 min for 40 cycles. The primer sequences used in this study were as follows: actin-forward, 5′-AACAGTCCGCCTAGAAGCAC-3′; actin-reverse, 5′-CGTTGACATCCGTAAAGACC-3′; Mal-forward, 5′-ATGCAGCCTACCACTGTGTG-3′; Mal-reverse, 5′-CAGCATGGACCACGTAGATC-3′; vilin-like (Vill)-forward, 5′-GAGGACCTAGGAGACCAGAC-3′; and Vill-reverse, 5′-ATTCTCCGCATCTACCACAG-3′.

### 2.7. Western Blotting

Western blotting was performed, as previously described [49]. In brief, cells were lysed with extraction buffer. Total proteins were separated after electrophoresis and were transferred onto an NC (nitrocellulose) membrane (pore size: 0.22 um; GE Healthcare). The intensities of the bands were recorded by a Tanon chemiluminescence detector (S5200, Tanon, Shanghai, China) after the signals were developed using the ECL (enhanced chemiluminescence) system (GE Healthcare). The protein levels of Mal and β-actin were indirectly detected using anti-Mal antibodies (ab15418, Abcam, Shanghai, China) and anti-β-actin antibodies (No. 3700, CST), respectively.

### 2.8. Histology

For histological analysis, subcutaneous tumors were fixed in PBS buffer containing 10% formaldehyde and were embedded in paraffin. Sections were stained with hematoxylin and eosin. The cell shape was observed under a microscope.

### 2.9. Actin Cytoskeleton Staining

Cells were seeded onto cover glasses and were allowed to grow to near 100% confluence. Cells were also seeded, allowed to grow to 100% confluence, and then subjected to a wound-healing assay. Cells were washed with PBS, fixed with 10% formaldehyde for 10 min, and permeabilized in 0.1% Triton X-100 for 3 min. Cells were then stained for 15 min with phalloidin-FITC (ab235137, Abcam). Photos were captured with an Olympus fluorescence microscope.

### 2.10. The Cancer Genome Atlas (TCGA) Data Mining and Gene Expression Correlation Analysis

For *MAL* and *VILL*, we obtained the fragments per kilobase of exon per million reads (FPKM) expression data and the related clinical data of patients with various types of cancer from The Cancer Genome Atlas (TCGA) database (https://www.proteinatlas.org, accessed on 1 September 2020). Patients were stratified into *MAL*- or *VILL*-high and *MAL*- or *VILL*-low groups. Survival curves were made with GraphPad Prism 7 software. Correlation analysis of the gene expression of *MAL* with that of other genes was performed using an online tool (http://www.linkedomics.org/login.php) [60].

### 2.11. Statistical Analysis

The *t*-test and log-rank test were performed using GraphPad Prism 7 software. The Spearman correlation test was performed using an online tool (http://www.linkedomics.org/login.php, accessed on 1 October 2020) [60]. Data were shown as the mean ± SEM. *p*-Values equal to or less than 0.05 were considered statistically significant. *n* denotes the number of experiments or samples.

## 3. Results

### 3.1. Runx3 Re-Expression Induces a Cell Shape Change and Suppresses the Migration and Metastasis of B16-F10 Melanoma Cells

**Figure 1:**
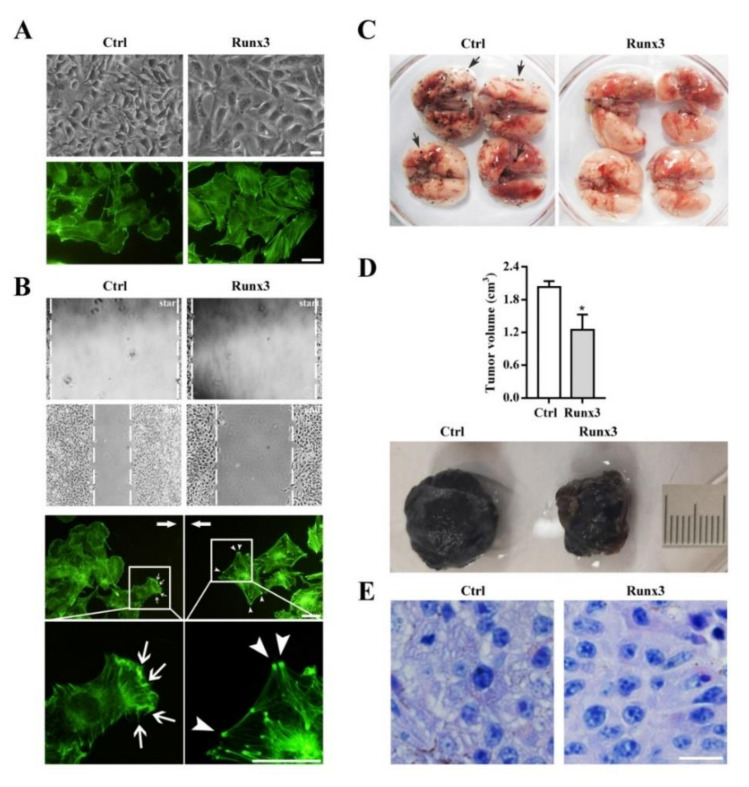
Runt-related transcription factor-3 (Runx3) re-expression in B16-F10 melanoma cells resulted in a cell shape change and suppressed the pulmonary metastasis of the cells. (**A**) Runx3 changed the cell shape and altered the actin cytoskeleton. The green fluorescent signals indicate actin filaments. (**B**) Runx3 delayed cell migration, and altered the dynamics of stress fiber formation in the migrating cells. *Start* denotes when the wound is just made, and *end* denotes when wound healing is terminated. The green fluorescent signals indicate actin filaments. The arrows indicate the directions of migration fronts. The arrowheads indicate the ends of stress fibers. The thin arrows indicate microspike-like structures. (**C**) Runx3 completely suppressed pulmonary metastasis. *n* = 4. The black dots on the surface of the lungs indicate metastasis foci. The windows-arrows representatively indicate metastasis foci. (**D**) Runx3 slightly reduced the tumor formation rate. The averaged tumor volumes are compared to each other (mean ± SEM, *n* = 3). * *p* < 0.05 is generated by the *t*-test. A representative image is shown. Bar: 1 cm. (**E**) Runx3 changed the cell shape in vivo. A representative section is shown. Ctrl: mock control B16-F10 cells; Runx3: B16-F10/Runx3 cells. All bars: 22 μm.

Melanocytes are derived from neural crest cells, and they are generally regarded as branched/dendritic pigment cells [61,62]. Melanoma has an origin in melanocytes [63]. Therefore, the cell shape of B16-F10 melanoma cells was described as elongated and branched when they were cultured in vitro [49]. Upon restoration of Runx3 expression, B16-F10 cells underwent a significant cell shape change from elongated and branched to spread and unbranched [49] (Figure 1A). In the meantime, stress fiber formation was considerably enhanced [49] (Figure 1A). In association with the cell shape change, migration was significantly attenuated [49] (Figure 1B and Figure S1). Interestingly, stress fibers in the migrating B16-F10/Runx3 cells degenerated, with the exception of their ends (Figure 1B as compared to Figure 1A). In contrast, along the migration front, some B16-F10 mock control cells showed microspike-like structures (Figure 1B as compared to Figure 1A), which were never formed in the Runx3-expressing B16-F10 cells (Table S1). Thus, it seemed that B16-F10/Runx3 cells entrapped themselves to at least slow down their migration. Further, although the B16-F10 mock control cells demonstrated a high potential of pulmonary metastasis, as expected [57], the metastatic potential was abolished by Runx3, whereas subcutaneous tumor formation was reduced by Runx3 to 61.5% compared to the mock control cells (Figure 1C,D). Meanwhile, the morphological differences between B16-F10/Runx3 and B16-F10 cells remained in vivo (Figure 1E).

### 3.2. Runx3 Re-Expression Alters a Specific Transcriptional Profile in B16-F10 Melanoma Cells

**Figure 2:**
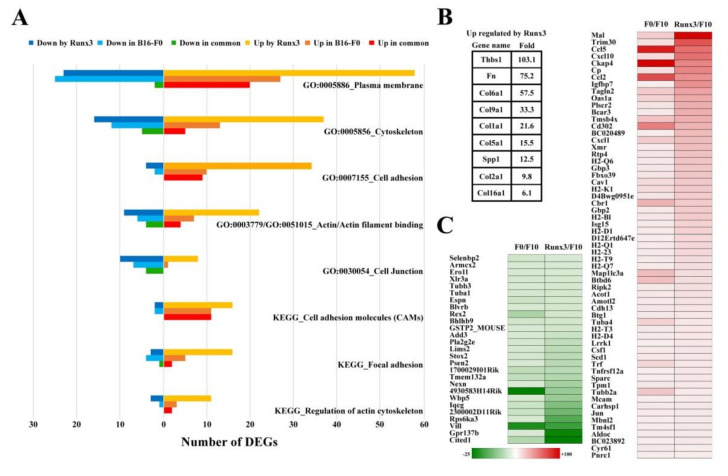
Microarray analysis uncovered an altered and specific transcriptional profile underlying the cell shape change and the suppression of the metastatic potential by Runx3. (**A**) Gene Ontology (GO) and Kyoto Encyclopedia of Genes and Genomes (KEGG) annotations assigned different repertoires of differentially expressed genes (DEGs) that were regulated by Runx3 in B16-F10 cells and, in the case of B16-F0 cells vs. B16-F10 cells, to the terms relevant to the actin cytoskeleton and adhesion. Each GO/KEGG annotation term is inscribed as a caption. The number of DEGs within a repertoire is described at x axis. The columns for the downregulated and the upregulated DEGs are shown in different colors as explained at the top of the figure., *Down/Up by Runx3* indicates the appearance of DEGs in the case of B16-F10/Runx3 cells vs. mock control B16-F10 cells. *Down/Up in B16-F0* indicates the appearance of DEGs in the case of B16-F0 cells vs. B16-F10 cells. *Down/Up in common* indicates the appearance of same DEGs in both cases. (**B**) Runx3 upregulated the expression of extracellular matrix (ECM) genes. The summary table indicates the gene names and their corresponding fold changes. (**C**) Runx3 regulated the expression of a list of DEGs that were inversely associated with an increase in the metastatic potential of B16-F10 cells compared to B16-F0 cells. The heatmap shows the gene names and their fold changes in the cases of B16-F10/Runx3 cells vs. mock control B16-F10 cells and B16-F0 cells vs. B16-F10 cells, respectively.

We performed microarray analysis for the identification of differentially expressed genes (DEGs) regulated by Runx3 re-expression in B16-F10 (high-metastatic) melanoma cells [57]. We also performed microarray analysis for the identification of DEGs in the comparison of B16-F0 (low-metastatic) melanoma cells with B16-F10 cells, since B16-F10 cells gained a high potential for pulmonary metastasis from B16-F0 cells via unknown genetic and/or epigenetic mechanisms [58]. In general, 783 DEGs and 499 DEGs were regulated in the case of Runx3 re-expression in B16-F10 cells and B16-F0 vs. B16-F10 cells, respectively. The differential expression of a few DEGs was confirmed previously and in this study for the validation of microarray data [49,64] (Figure S2). With an emphasis on the aforementioned cell shape change and its associated attenuation of the migratory and metastatic potential, we performed molecular annotation analysis of the microarray data using the GO database and the Kyoto Encyclopedia of Genes and Genomes (KEGG) database. According to the GO_cellular component/biological process/molecular function annotations, we found in both cases that the various repertoires of DEGs were assigned to the terms of GO:0005886-plasma membrane, GO:0005856-cytoskeleton, GO:0007155-cell adhesion, GO:0003779/0051015-actin/actin filament binding, and GO:0030054-cell junction (Figure 2A). According to the KEGG_pathway annotation, we found in both cases that the different repertoires of DEGs were assigned to the terms cell adhesion molecules (CAMs), focal adhesion, and regulation of the actin cytoskeleton (Figure 2A).

Notably, we found that Runx3 specifically upregulated a number of DEGs that encode major ECM proteins in B16-F10 cells, some of which have been reported to play key roles in focal adhesion formation [23,24,65] (Figure 2B).

Interestingly, among the upregulated DEGs in the case of B16-F0 vs. B16-F10 cells, 61 were also found to be upregulated by Runx3 in B16-F10 cells; among the downregulated DEGs in the case of B16-F0 vs. B16-F10 cells, 27 were also downregulated by Runx3 in B16-F10 cells, indicating that there was a commonly altered transcriptional profile in both cases (Figure 2C). In addition, 10 CAM-encoding DEGs (*H2-K1*, *H2-Bl*, *H2-Q6*, *H2-Q7*, *H2-Q1*, *H2-D1*, *H2-T3*, *H2-T9*, *H2-T23*, and *Mcam*) were upregulated in both cases (Figure 2C); indeed, downregulation of murine CAMs has frequently been found in metastatic tumors [66].

### 3.3. The Runx3-Altered Transcriptional Profile May Have Prognostic Value for Various Cancers, as Evidenced by MAL and/or VILL

**Figure 3:**
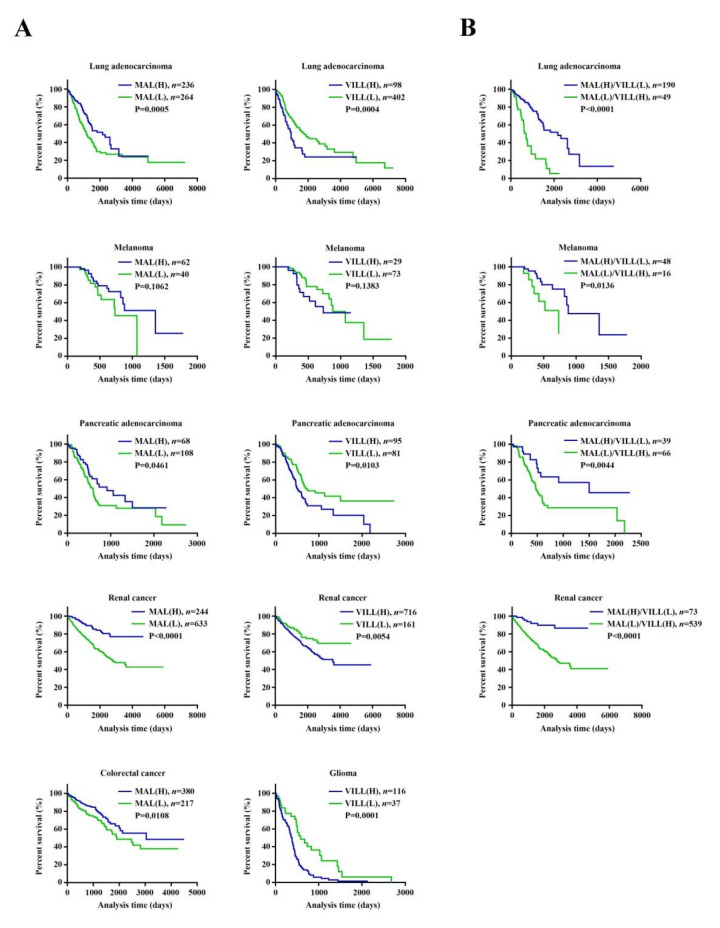
The gene expression of myelin and lymphocyte protein (*MAL*) and/or vilin-like (*VILL*) had prognostic value for various cancers. (**A**) Kaplan–Meier survival analysis implicated *MAL* or *VILL* in various cancers. (**B**) Kaplan–Meier survival analysis implicated both *MAL* and *VILL* in various cancers. The information about cancer type, patient number, and *p*-value is inscribed at each panel. The gene level was defined as fragments per kilobase of exon per million reads (FPKM). The median cutoff was used to group patients into low (L) and high (H) expression of *MAL* and/or *VILL*. The *p*-values are generated by the log-rank test.

To address the clinical relevance of the Runx3-altered transcriptional profile, we selected *Mal* and *Vill* from the list of commonly altered DEGs to perform data mining using the gene expression data and the corresponding clinical parameters that were available from The Cancer Genome Atlas (TCGA). In Kaplan–Meier survival analysis, patients were stratified into *MAL*-/*VILL*-high and *MAL*-/*VILL*-low groups for each type of cancer. MAL has previously been reported to be a metastasis suppressor in esophageal cancer, gastric cancer, and head and neck squamous cell carcinoma and seems to confer survival advantages for relevant patients [53,56,67,68]. We demonstrated here that high-*MAL* gene expression seems to confer survival advantages for patients with melanoma, lung adenocarcinoma, pancreatic adenocarcinoma, renal cancer, or colorectal adenocarcinoma (Figure 3A). VILL’s role in metastasis is unclear. We demonstrated that low-*VILL* gene expression seems to confer survival advantages for patients with melanoma, lung adenocarcinoma, pancreatic adenocarcinoma, renal cancer, or glioma (Figure 3A). Notably, we demonstrated that both high-*MAL* gene expression and low-*VILL* gene expression seem to synergistically confer survival advantages for patients with melanoma, lung adenocarcinoma, pancreatic adenocarcinoma, or renal cancer (Figure 3B). We also performed Kaplan–Meier survival analysis for *RUNX3* within the same types of cancer. We demonstrated that *RUNX3* does not have prognostic value or a positive prognostic value, even though it is usually regarded as a tumor suppressor gene (Figure S3).

### 3.4. Mal Gene Expression Is Correlated with Metastatic Potential among Cellular Models and Is Subject to Epigenetic Regulation

**Figure 4:**
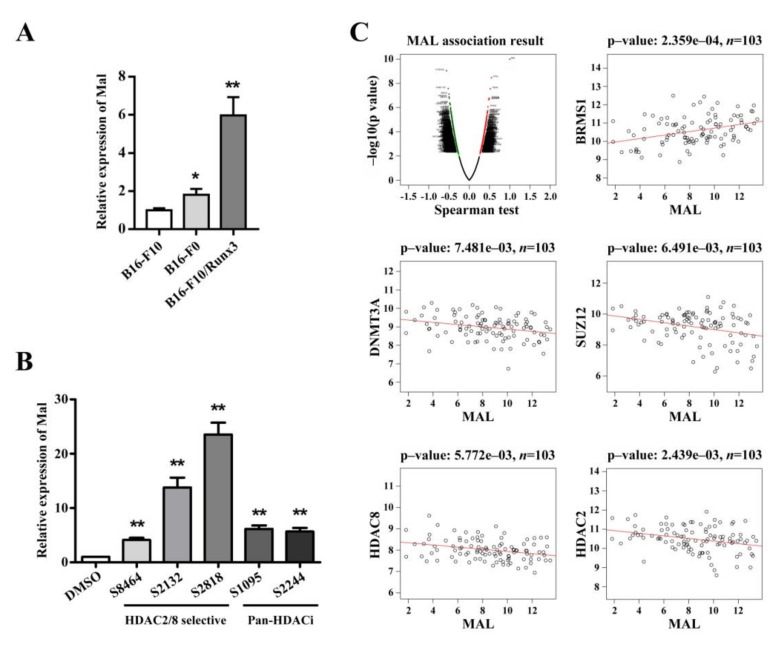
*Mal* gene expression was correlated with metastatic potential among the cell lines and was subject to epigenetic regulation. (**A**) *Mal* gene expression was validated in the cells used in this study with qRT-PCR. The relative *Mal* gene expression levels were compared among the indicated cell lines (means ± SEM, *n* = 3). (**B**) Treatment with histone deacetylase (HDAC) inhibitors induced the gene expression of *Mal* in B16-F10 cells. The relative *Mal* gene expression levels of inhibitor-treated cells were compared to those of untreated (DMSO) cells (means ± SEM, *n* = 3). (**C**) The gene expression of *MAL* was correlated with that of other genes in human melanoma. The top left panel indicates the overall *MAL* correlation. The other panels indicate the correlation between *MAL* and the other genes. The information about patient number and *p*-value is inscribed at each panel. The gene level at *x*/*y* axis is defined from FPKM. The *p*-values are generated by the Spearman correlation test. * *p* < 0.05 and ** *p* < 0.01 are generated by the *t*-test.

Based on our microarray data and the results of our survival analysis, we selected *Mal* for further investigation. We validated the gene expression of *Mal* in B16-F10, B16-F0, and B16-F10/Runx3 cells. As shown in Figure 4A, both B16-F10/Runx3 cells and B16-F0 cells had an increased level of *Mal* mRNA compared to B16-F10 cells. As Runx3 suppressed metastasis of B16-F10 cells and B16-F0 cells are known to have a lower metastatic potential than B16-F10 cells, we believe that the level of *Mal* gene expression is correlated with the metastatic potential among the melanoma cells used in this study.

To address the pharmaceutical regulation of *Mal* gene expression, we randomly selected five HDAC inhibitors; two of these molecules are pan-HDAC inhibitors, and the others are HDAC2/8-selective inhibitors. We found that these HDAC inhibitors dramatically induced *Mal* gene expression in B16-F10 cells (Figure 4B), suggesting that *Mal* gene expression is subject to epigenetic regulation.

We also performed correlation analysis between the gene expression of *MAL* and that of other genes in melanoma (Figure 4C). We found that *MAL* was positively correlated with a known metastasis suppressor, *BRMS1* [7] (Figure 4C). We also found that *MAL* was negatively correlated with a few genes involved in de novo methylation of genomic DNA or deacetylation of histones (Figure 4C).

### 3.5. Mal Is Necessary for Suppressing the Migratory and Invasive Traits of Metastasis in Melanoma Cells

**Figure 5:**
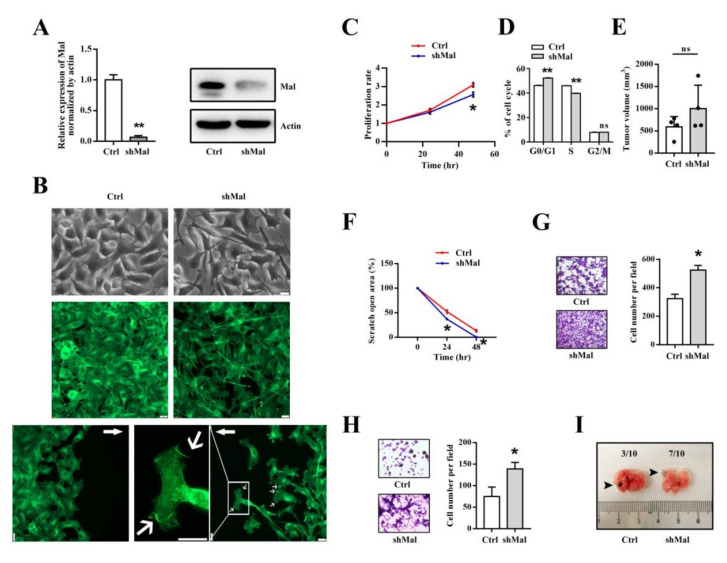
Mal was necessary for suppressing the migratory and invasive traits of metastatic melanoma cells. (**A**) The knockdown of *Mal* gene expression was validated with qRT-PCR and Western blotting. The relative *Mal* gene expression levels are compared to each other (means ± SEM, *n* = 3). Mal’s molecular weight: 18 kDa; actin’s molecular weight: 43 kDa. (**B**) The knockdown of *Mal* gene expression changed the cell shape and altered the actin cytoskeleton of steady or migrating cells. The green fluorescent signals indicate actin filaments. The arrows indicate the directions of migration fronts. The thin arrows indicate microspike-like structures. Bars: 22 μm. (**C**) The knockdown of *Mal* gene expression slightly reduced the proliferation rate. The relative proliferation rates were determined by 3-(4,5-dimethyl-2-thiazolyl)-2,5-diphenyl-2-H-tetrazolium bromide (MTT) and were compared to each other (means ± SEM, *n* = 3). (**D**) The knockdown of *Mal* gene expression slightly delayed the progress of the cell cycle. The percentages of cells at various phases (G0/G1, S, and G2/M) were determined by flow cytometry (means ± SEM, *n* = 3, ns: not significant). (**E**) The knockdown of *Mal* gene expression had no effect on the tumor formation rate. The averaged tumor volumes were compared to each other (means ± SEM, *n* = 4, ns: not significant). (**F**) The knockdown of *Mal* gene expression increased the migration rate in wound healing. The relative scratch open areas were compared to each other (means ± SEM, *n* = 3). (**G**) The knockdown of *Mal* gene expression increased the migration rate through the Transwell chambers. A representative image is shown on the left of the panel, and the averaged cell numbers were compared to each other, as shown on the right (means ± SEM, *n* = 6). (**H**) The knockdown of *Mal* gene expression increased the invasion rate through Matrigel-coated Transwell chambers. A representative image is shown on the left of the panel, and the averaged cell numbers were compared to each other, as shown on the right (means ± SEM, *n* = 6). (**I**) The knockdown of *Mal* gene expression increased the frequency of metastasis-focus-positive lungs. A representative image is shown. The arrowheads indicate metastasis foci. The frequencies are inscribed at the top of the image. Ctrl: mock control; shMal: short hairpin RNA interference of *Mal*. * *p* < 0.05 and ** *p* < 0.01 are generated by the *t*-test.

To investigate the functional roles of Mal in melanoma, we lentivirally reduced the gene expression of *Mal* in B16-F0 cells (Figure 5A). We found that the knockdown of *Mal* gene expression apparently resulted in a cell shape change from spread to elongated cells (Figure 5B, Figure S4). In line with the cell shape change, the knockdown of *Mal* gene expression altered the actin cytoskeleton and, notably, induced the formation of microspike-like structures in some cells along the migration front (Figure 5B). Meanwhile, the knockdown of *Mal* gene expression slightly reduced the proliferation rate, as shown by the altered progress of the cell cycle, but had no effect on the subcutaneous tumor formation rate (Figure 5C–E). However, it significantly enhanced the migratory and invasive potential (Figure 5F–H). Unfortunately, we found that the knockdown of *Mal* gene expression did not dramatically increase the number of metastasis foci in the lungs (Figure 5I). Instead, it increased the frequency of metastasis-focus-positive lungs (Figure 5I).

## 4. Discussion

The search for therapeutic targets for metastasis has been a continuous journey [1,7,8,9,14,20,22,69,70]. Targeting cancer cell motility can be an effective strategy for metastasis therapy because cell motility is a key driving trait of metastasis and is required for multiple stages of metastasis at least via adhesion and the actin cytoskeleton [1,20]. In addition, increases in the metastatic potential of melanoma cells could be associated with cell shape change [13], a phenomenon that has already been noticed [26,57]. In this study, by using cellular models of mouse melanoma, we uncovered a specific Runx3-regulated transcriptional profile that contained DEGs that encoded some ECM proteins, as well as DEGs that were inversely associated with increases in the metastatic potential. This profile was correlated with the phenotype of the cell shape change and the attenuation of migration and metastasis, with comprehensive annotations referring to the actin cytoskeleton and adhesion. The DEGs were identified using microarray data of mouse melanoma, but they can be interpreted in future clinical studies, as evidenced by others [71]. Thus, we suggest that the self-entrapping of cells via adhesion and the actin cytoskeleton could be a powerful strategy for metastasis therapy for melanoma.

Whether Runx3 is a metastasis suppressor in melanoma is an important issue. First, it was reported that Runx3 expression is frequently lost in melanoma and that there is a good negative correlation between Runx3 expression and melanoma malignancy [40]. Second, we found that Runx3 re-expression abolishes the pulmonary metastasis of B16-F10 cells, but it slightly affects the subcutaneous tumor formation rate (Figure 1C,D). This evidence is in accordance with the functional definition of a metastasis suppressor by Khan and Steeg [7]. Third, and most importantly, we found that Runx3 can regulate a specific profile of DEGs that is inversely associated with an increase in the metastatic potential of B16-F10 cells compared to B16-F0 cells (Figure 2C).

It is a distinct feature that Runx3 re-expression in B16-F10 cells upregulates *fibronectin* (*Fn*) and the other major ECM genes without downregulating any other ECM genes (Figure 2B). This feature may implicate a metastasis-suppressing role played by Fn, even though Fn is usually regarded as an EMT marker in many cancer studies. In fact, early studies suggested that the loss of Fn is correlated with the acquisition of metastasis [72]. Recently, the role of Fn in protecting against the EMT process was confirmed in vivo [73]. In addition, the metastatic potential seems to be strongly related to the binding capacity of the specific constituent combination of Fn with other ECM molecules [34]. Therefore, the roles of ECM proteins in metastasis should be considered carefully.

Several previous reports have demonstrated that Mal is a tumor suppressor [53,55]. Mal is a four-time transmembrane protein; in particular, it is a lipid raft constituent [53], and it could be a therapeutic target because of its features as a cell surface protein [14]. Mal is thus a participant in RhoA signaling and has a role in stress fiber formation and focal adhesion formation [54]. Mal expression in esophageal cancer cells suppresses migration and invasion [67]. Suppression of Mal expression in gastric cancer is correlated with metastasis [68]. Downregulation of Mal expression may contribute to the metastatic potential of head and neck squamous cell carcinoma [53,56]. In this study, it was demonstrated that Mal is necessary for suppressing the migratory and invasive traits of metastasis in mouse melanoma cells; its expression can be of prognostic value and can be subject to epigenetic regulation. Since transcription factors are hard to target in cancers, we suggest that Mal can be a therapeutic target for the treatment of metastasis of melanoma cells.

Like *MAL*, *VILL* gene expression also has a prognostic value, and an increase in *MAL* gene expression and/or a decrease in *VILL* gene expression may confer survival advantages in several types of cancer. In view of making use of *MAL* and *VILL* for the treatment of metastasis of these types of cancer, one can use small molecules to enhance *MAL* gene expression or function and/or to attenuate *VILL* gene expression or function. Our result shows that *Mal* gene expression would be pharmaceutically enhanced by HDAC inhibitors in metastatic mouse melanoma cells. Moreover, this result implicates that there could be an HDAC-mediated epigenetic mechanism when B16-F10 cells gain high potential for pulmonary metastasis compared to B16-F0 cells. Further studies are needed to address whether there are HDAC-mediated epigenetic mechanisms in human melanoma. If there exists such a mechanism that could be implicated in melanoma metastasis, a specific HDAC inhibitor could be useful in the future for the treatment of metastasis of melanoma cells.

## Data Availability

The raw microarray data and the processed microarray data can be viewed at https://www.ncbi.nlm.nih.gov/geo/query/acc.cgi?acc=GSE161991 and https://www.ncbi.nlm.nih.gov/geo/query/acc.cgi?acc=GSE161992.

## Supplementary Materials

The following are available online at https://www.mdpi.com/1422-0067/22/4/2219/s1: Figure S1: Runx3 expression in B16-F10 melanoma cells delayed the migration rate in wound healing (quantification); Figure S2: The *Vill* gene expression was validated in the cell lines used in this study with qRT-PCR; Figure S3: The gene expression of *RUNX3* had no prognostic/positive prognostic value for various cancers; Figure S4: The knockdown of *Mal* gene expression changed the cell shape (at subconfluence); and Table S1: Frequency of microspike-like structures per 10 random views.

## Abbreviations

DEG	differentially expressed gene
ECM	extracellular matrix
EMT	epithelial–mesenchymal transition
FPKM	fragments per kilobase of exon per million reads
GO	Gene Ontology
HDAC	histone deacetylase
KEGG	Kyoto Encyclopedia of Genes and Genomes
Mal	myelin and lymphocyte protein
PBS	phosphate-buffered saline
qRT-PCR	quantitative reverse transcription–polymerase chain reaction
Runx3	runt-related transcription factor-3
TCGA	The Cancer Genome Atlas
Vill	vilin-like
