# Supplementary material for: Runx3 Induces a Cell Shape Change and Suppresses Migration and Metastasis of Melanoma Cells by Altering a Transcriptional Profile
Source: Int J Mol Sci. 2021 Feb 23;22(4):2219. doi: 10.3390/ijms22042219 (PMC7926509; doi:10.3390/ijms22042219)
Supplement: Supplementary file 1 [file ijms-22-02219-s001.zip › Supplementary figure legends.docx]

**Figure S1.** Runx3 expression in B16-F10 melanoma cells delayed the migration rate in wound healing (quantification). The averaged scratch open areas are compared to each other (means ± SEM, *n* = 2). * (*p* = 0.05) is generated by the *t* test. The original figure supporting for the graph is shown below. *Start* denotes when wound is just made, and *end* denotes when wound healing is terminated. Ctrl: mock control B16-F10 cells, Runx3: B16-F10/Runx3 cells.

**Figure S2.** The *Vill* gene expression was validated in the cell lines used in this study with qRT-PCR. The relative *Vill* gene expression levels, which are normalized by actin, are compared among the indicated cell lines (means ± SEM, *n* = 3). ** (*p* < 0.01) is generated by the *t* test.

**Figure S3.** The gene expression of *RUNX3* had no prognostic/positive prognostic value for various cancers. The Kaplan–Meier survival analysis implicated *RUNX3* in various cancers. The information of cancer type, patient number and *p* value is inscribed at each panel. The gene level is defined as FPKM. Median cutoff is used to group patients into the low (L) and the high (H) expression of *RUNX3*. The *p* values are generated by the log-rank test.

**Figure S4.** The knockdown of *Mal* gene expression changed the cell shape (at subconfluence). The cell-shape pictures were captured when cells were at subconfluence. Bar: 22 μm.
