# Supplementary material for: Runx3 Induces a Cell Shape Change and Suppresses Migration and Metastasis of Melanoma Cells by Altering a Transcriptional Profile
Source: Int J Mol Sci. 2021 Feb 23;22(4):2219. doi: 10.3390/ijms22042219 (PMC7926509; doi:10.3390/ijms22042219)
Supplement: Supplementary file 1 [file ijms-22-02219-s001.zip › Table S1.docx]

**Table S1.** Frequency of microspike-like structures per 10 random views

| B16-F10 mock control | B16-F10/Runx3 |
| --- | --- |
| 10/10 | 0/10 |
