# Supplementary figures and images for: Runx3 Induces a Cell Shape Change and Suppresses Migration and Metastasis of Melanoma Cells by Altering a Transcriptional Profile
Source: Int J Mol Sci. 2021 Feb 23;22(4):2219. doi: 10.3390/ijms22042219 (PMC7926509; doi:10.3390/ijms22042219)

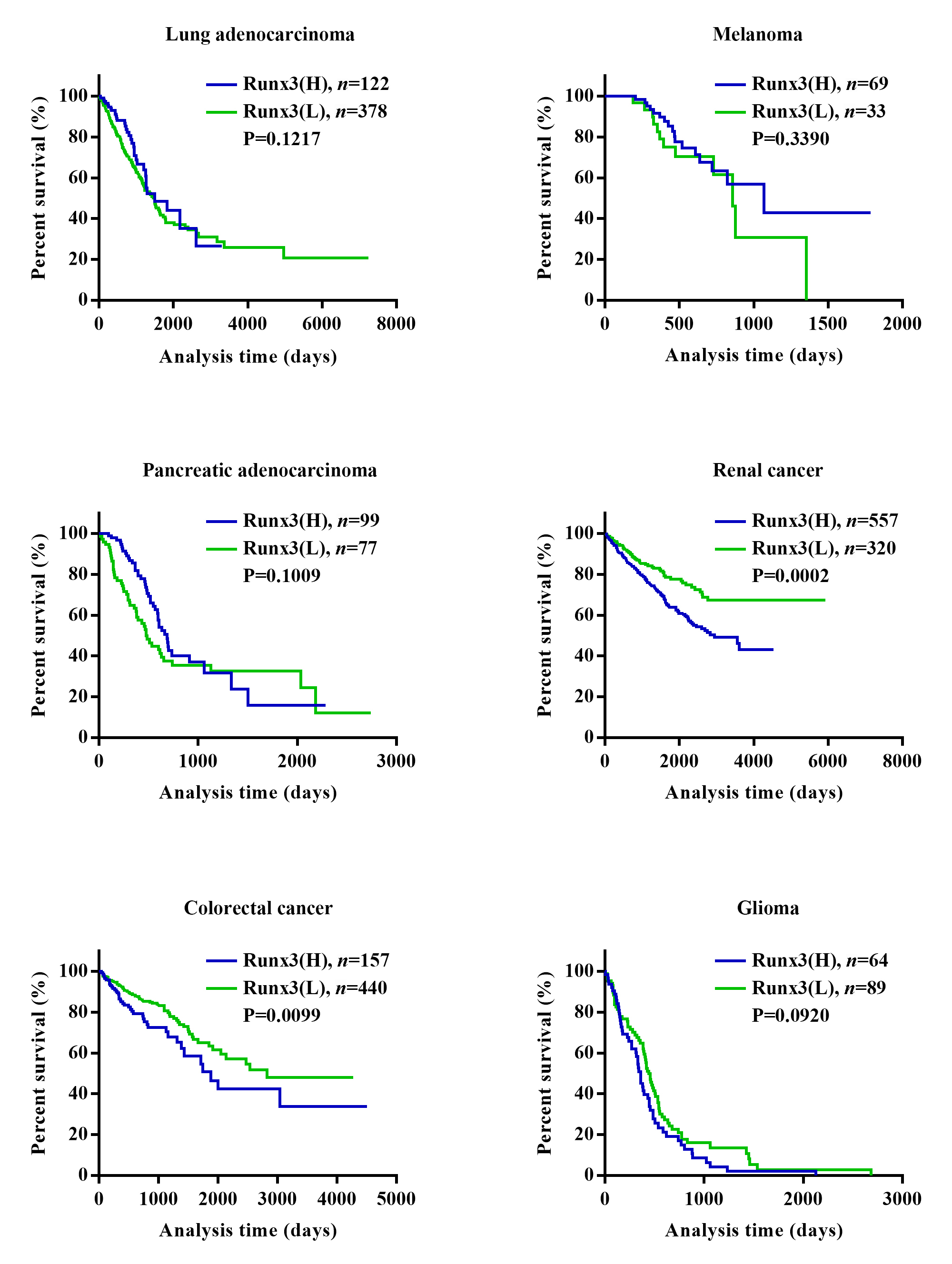

Supplement: Supplementary file 1 [file ijms-22-02219-s001.zip › Figure S3.jpg]

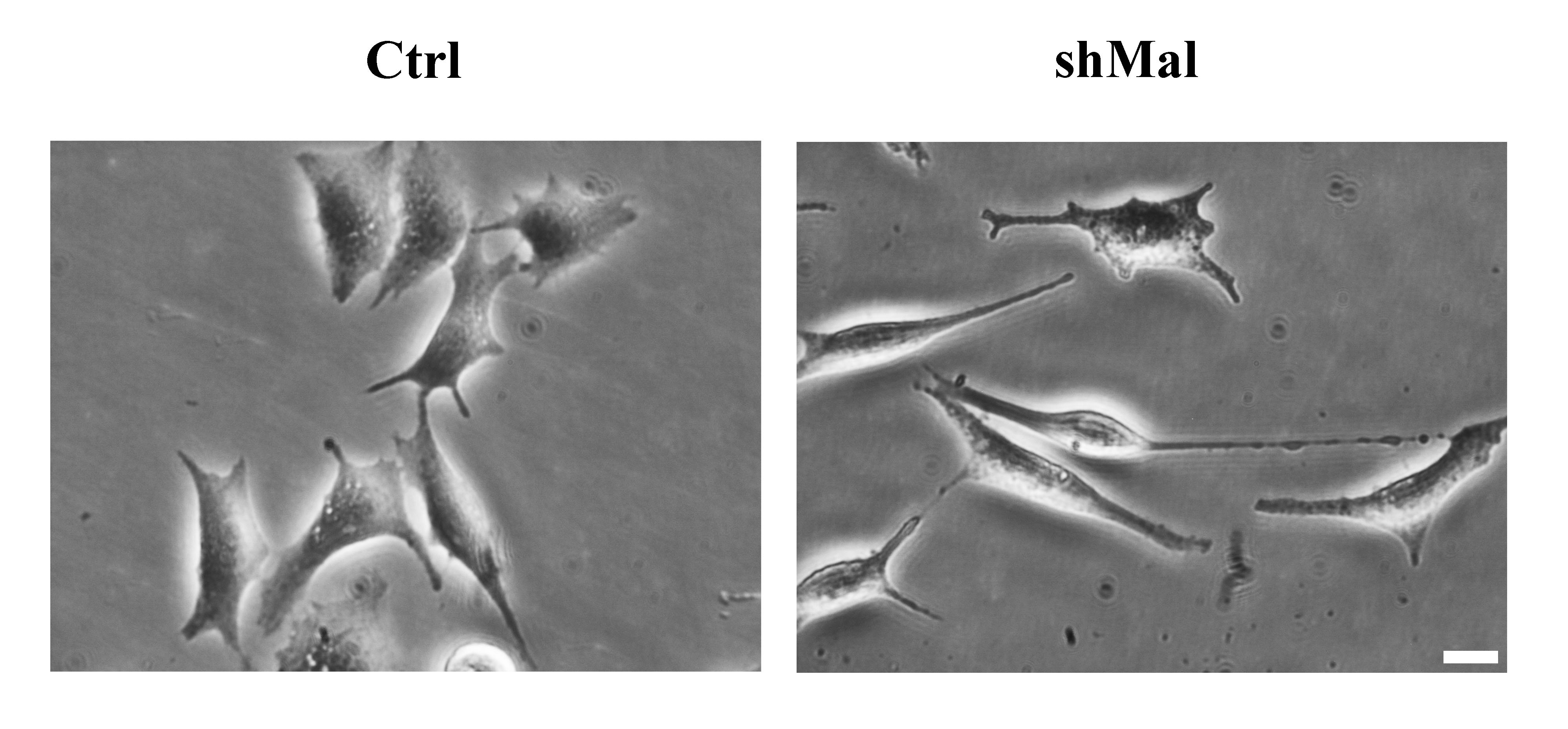

Supplement: Supplementary file 1 [file ijms-22-02219-s001.zip › Figure S4.jpg]

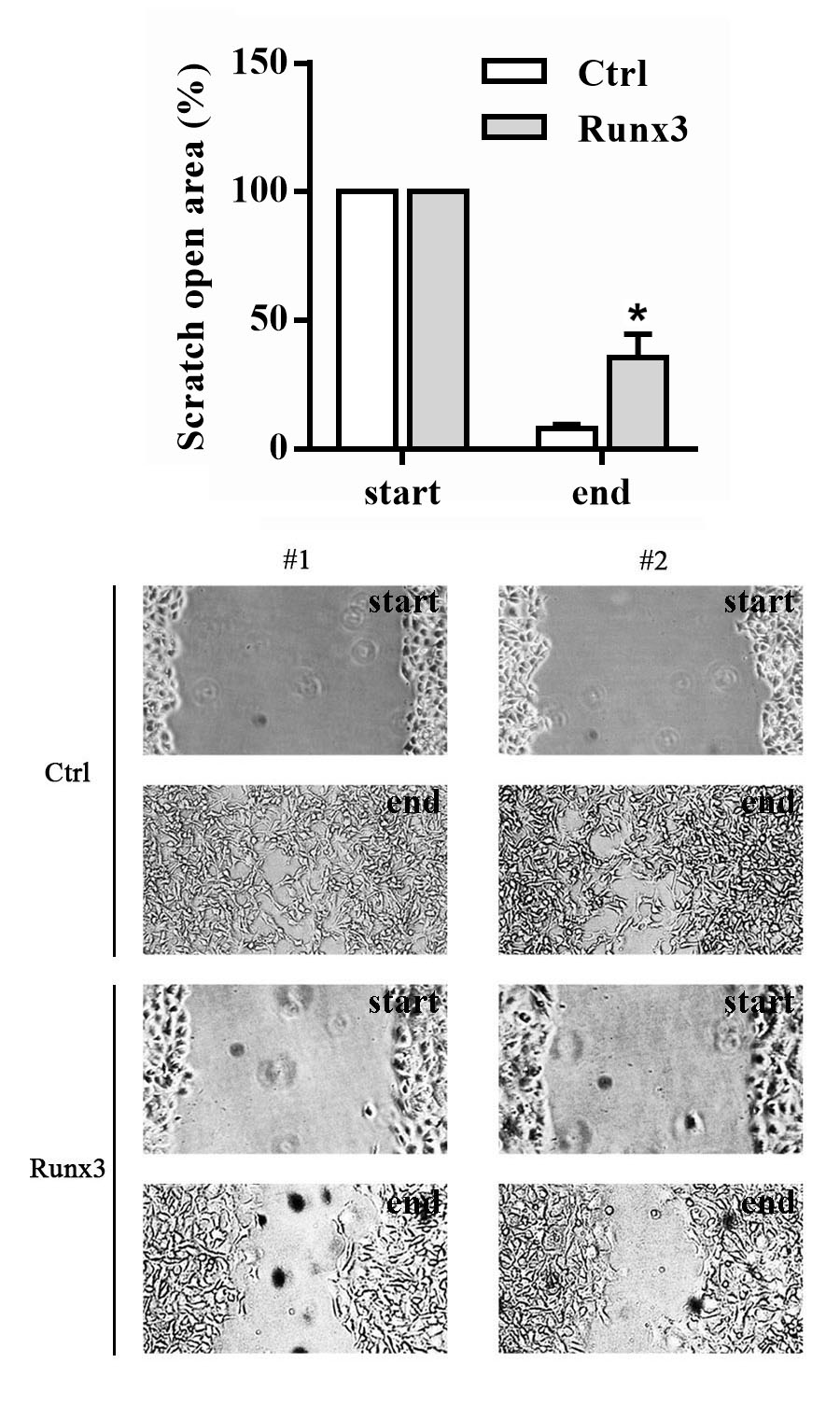

Supplement: Supplementary file 1 [file ijms-22-02219-s001.zip › Figure S1.jpg]

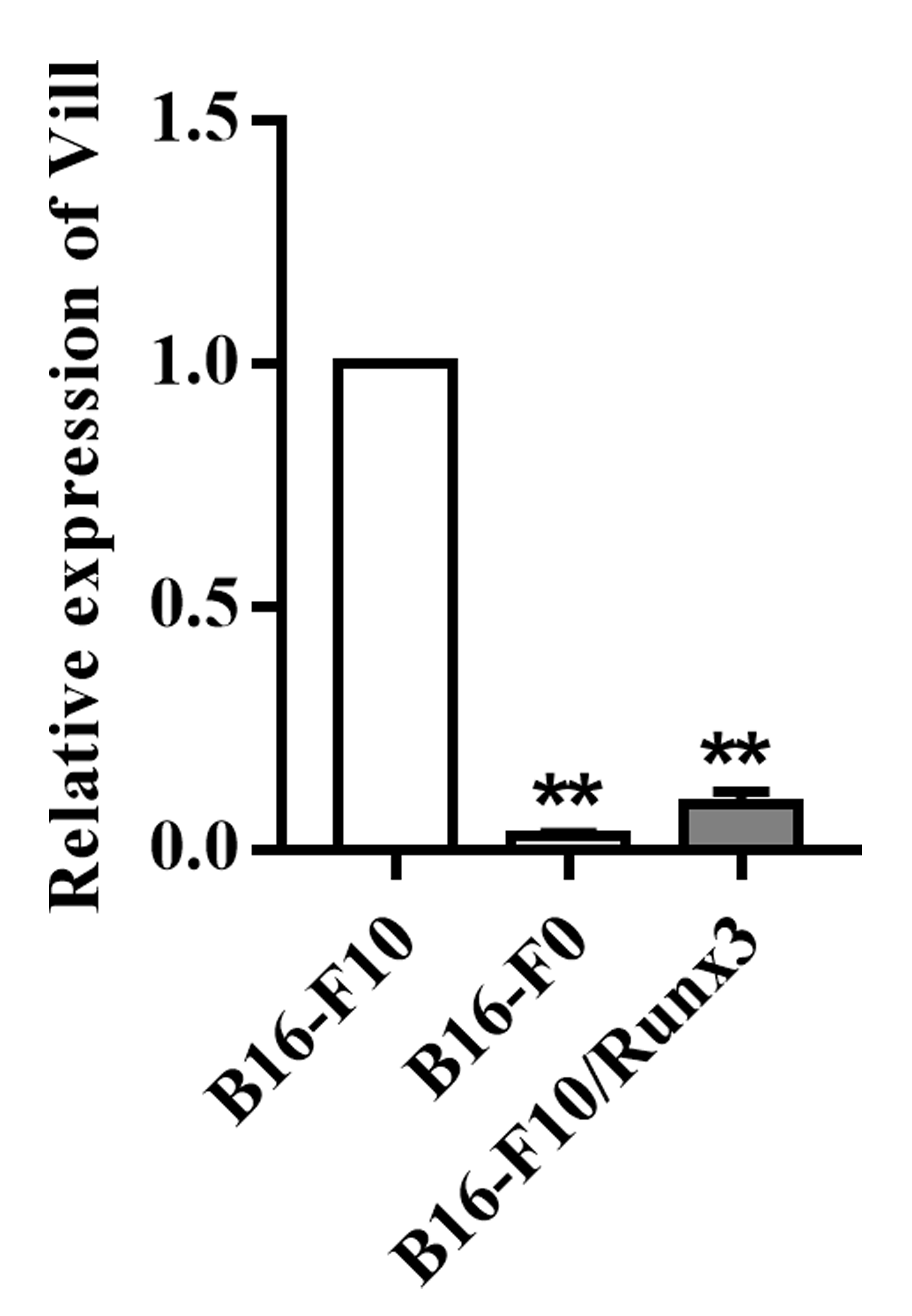

Supplement: Supplementary file 1 [file ijms-22-02219-s001.zip › Figure S2.jpg]
